# “You get exposed to a wider range of things and it can be challenging but very exciting at the same time”: enablers of and barriers to transition to rural practice by allied health professionals in Australia

**DOI:** 10.1186/s12913-020-4954-8

**Published:** 2020-02-10

**Authors:** Saravana Kumar, Esther Jie Tian, Esther May, Rosanne Crouch, Mandy McCulloch

**Affiliations:** 1 School of Health Sciences, University of South Australia, City East Campus, GPO Box 2471, Adelaide, SA 5001 Australia; 2 Division of Health Sciences, University of South Australia, City East Campus, GPO Box 2471, Adelaide, SA 5001 Australia; 3 Department of Rural Health, University of South Australia, City East Campus, GPO Box 2471, Adelaide, SA 5001 Australia; 4 Rural Doctors Workforce Agency, 63 Henley Beach Road, Mile End, SA 5031 Australia

**Keywords:** Transition, Allied health professional, Rural, Remote, Qualitative

## Abstract

**Background:**

There is consistent evidence highlighting the mal-distribution of the health workforce between urban and rural and remote regions. To date, addressing this mal-distribution has focused on medicine and nursing with limited initiatives targeted at allied health. Therefore, the aim of this research was to explore the enablers of and barriers to transition to rural practice by allied health professionals across South Australia in Australia.

**Method:**

Qualitative descriptive methodology was used to underpin this research. Individual, in-depth semi-structured interviews were conducted with employers, managers and allied health professionals from rural regions of South Australia who were identified using purposive maximum variation sampling strategy.

**Results:**

A total 22 participants shared their perspectives on the enablers of and barriers to transition to rural practice by allied health professionals across South Australia. Thematic analysis of the interview data resulted in a number of key issues impacting transition to rural-based practice. These findings could be broadly categorised into three stages during the transition: ‘before’; ‘during’ and ‘after’.

**Discussion:**

This study identified a range of enablers of and barriers to transition to rural practice by allied health professionals. Five overarching themes – nature of rural practice, exposure to rural ‘taster’, social/lifestyle, job availability/characteristics, and mentor and support were identified. In particular, exposure to rural ‘taster’, social/lifestyle, and mentor and support were the key themes reported by the stakeholders. The multifactorial nature of the barriers and enablers highlight the complexity underpinning how AHPs transition to rural-based practice. These barriers/ enablers are often inter-linked and continually evolving which pose significant challenges for health care stakeholders to successfully addressing these.

**Conclusion:**

This research sheds light on the complexities that confront and successful strategies that are required for health care stakeholders when considering how best to support allied health professional transition to rural practice.

## Introduction

Health care inequalities remain a significant issue for people living in rural and remote communities [1]. There is consistent evidence to indicate that people in these communities’ experience poorer health compared to their metropolitan counterparts [1–3] including, higher chronic disease and mortality rates [4]. When compared to city locations, mortality rates are 1.05–1.15 [5] and 1.3 higher [4] in regional and remote (and very remote) areas respectively. Additionally, risk of cardiovascular disease is significantly higher in rural areas with ischaemic heart disease death rates 1.2 (women) and 1.3 (men) times higher compared to metropolitan areas [6].

Causes of health inequalities are complex and often intertwined [7]. They include a complex mix of social economic factors including income, education and employment opportunities [8]. Access to health care services and providers are often limited [9] which also contributes to the poor health care outcomes of people living in rural and remotes areas [3]. Given these unique issues, addressing rural health disadvantage is complex and requires careful planning to meet the unique needs of rural and remote areas [10]. In response to this, many initiatives have been trialled including discrete, integrated, comprehensive Primary Health Care, outreach and virtual outreach services (e.g. telehealth). Many aimed to improve access to services and general practitioners [1].

Another means of tackling rural health disadvantage has been to increase supply of health care workforce to rural and remote areas [3, 11]. A recent inquiry reported that there were approximately 50 programs in Australia designed to help address the shortages of doctors in rural areas [12]. However, supply shortage is not unique to the medical and nursing professions. In fact, mal-distribution of the allied health professionals (AHPs) follows a similar pattern to doctors, with a decrease in AHPs with increasing remoteness [12]. For example only 0.8% of psychologists practice in rural areas, compared to 79.5% in metropolitan areas [12]. This shortage is particularly alarming considering the pivotal role AHPs play in the provision of health care services, such as chronic disease management, rehabilitation and acute care [13]. Addressing the mal-distribution and lack of AHPs in rural areas is of critical importance to help improve health inequalities experienced by Australians living in rural and remote locations [10].

To date, much of the focus on addressing health workforce shortages in rural and remote Australia has had a focus on medical and nursing professions [13]. In South Australia, while the Rural Health Professionals Program (RHPP) and the Transition to Professional Practice Program (TPPP) provide financial and professional supports to all health professionals including allied health, the uptake of these programs are mostly associated with the nursing professions. Currently there are few studies which have explored AHPs transition to rural practice. In a literature review, Campbell and colleagues [14] explored factors which influence recruitment and retention of AHPs in rural and remote areas. This review identified a range of extrinsic (such as remuneration packaging and professional development supports) and intrinsic motivation incentives (such as professional autonomy and feelings derived from work). The findings from this review highlighted that a significant number of extrinsic factors appear to have a negative influence on recruitment and retention, whereas majority of intrinsic factors impact on AHPs positively. The authors suggested that a balance of both extrinsic and intrinsic motivation incentives needs to be addressed to improve workplace shortages in rural areas.

Given the paucity of research on transition to rural practice by AHPs, the aim of this research was to explore the enablers of and barriers to transition to rural practice by AHPs across South Australia in Australia.

## Methods

### Methodology

Given the limited research on enablers of and barriers to transition to rural practice by AHPs, a qualitative descriptive (QD) research methodology [15] was used to explore this issue. The QD research methodology helps to obtain an accurate portrayal of the phenomena of interest by producing findings that are close to the collected data and within an identifiable local context [15]. This is achieved by descriptions of people characteristics, traits and behaviours that occur in everyday context using common language. As the focus of this research was to explore various barriers and enablers that confront AHPs within the South Australian rural practice setting, which can then be used to inform current practice and areas for future research, QD provided the ideal methodology to underpin this research.

### Study participants and selection procedures

A total of 52 potential participants (including 45 AHPs and seven employers/managers) practicing within various rural and remote areas across South Australia were identified through key allied health contacts and invited to participate via emails initially, which were then followed up by direct phone contact. The research team contacted these AHPs and employers/managers from a broad range of experience and disciplines, years of experience and diversity of roles. Inclusion criteria were AHPs who had recently transitioned to rural practice and employers from rural-based organisations. Participants were purposefully identified through their past participating in programs such as RHPP, the TPPP, and through agencies which provided access to these programs (such as the Rural Doctors Workforce Agency (RDWA)). For the purpose of this research, the term rural and remote encompassed all areas outside of South Australia’s major city (Adelaide) and included Barossa Valley, Eyre Peninsula. Far North, Fleurieu Peninsula, Flinders Ranges, Kangaroo Island, Limestone Coast, Mid North, Murray Mallee and Yorke Peninsula.

Considerations for sampling and sample size in qualitative research are not focused on representation of the population but rather on methodological and practical considerations [16]. Methodological consideration includes aspects such as data saturation (when data collection gathered no new information), variability within the sample and opportunities to explore in-depth issues at hand. Practical consideration includes availability of resources, costs and time required to undertake data collection and analysis [16]. Based on the methodological and practical considerations, participants for this research were recruited using purposive maximum variation sampling strategy [17, 18] and data collection continued until such time that no new information was observed in the data. This sampling strategy was particularly chosen as it helps to identify diverse variations and maps common patterns that may exist across variations.

### Data collection

All data was collected via individual, semi-structured interviews, led by members of the research team (SK [an experienced, senior male researcher who was a physiotherapist by training and had extensive expertise in allied health practice and research] and ET [a female researcher who was a dietitian by training with a growing expertise in allied health practice and research]). This method of data collection was chosen as it assists in gaining in-depth and independent understanding of participant’s point of view [19]. An interview guide was developed in consultation with key stakeholders and piloted within the research team, who were all trained as allied health professionals [20]. The questions were broadly based on barriers and facilitators specific to the transition to rural-based practice. Questions were generally focused on what worked, what didn’t and what, if any improvements, could be made to improve transition. It consisted of a comprehensive set of open-ended questions which directed the interview broadly, with opportunities for prompts [18]. By ensuring these questions were open-ended, this method assisted in minimizing bias in participants responses. During the conduct of the interview, researchers were able to seek additional clarifications and participants elaborated further. Each interview lasted approximately 30–45 min and was conducted via telephone in a secure office. All interviews were audio-recorded and transcribed verbatim.

### Data analysis

As methodology underpinning this research was QD, content and thematic analysis are two types which are commonly used [16]. Both content and thematic analysis share some similarities as both identify common patterns and themes. In this instance thematic analysis was chosen to analyse the data [18]. The coding process was discussed and trialled within the research team (using one transcript) prior to the conduct of data analysis to ensure a consistent approach. Once this had been established, the transcripts were divided between the research team and coding process began manually. Each interview transcript was read independently by the researcher and ideas generated from this process were labelled as codes. The same process was repeated across multiple transcripts and common codes were identified and categorised to form themes [17, 19]. These themes were then labelled according to messages that they represented. A third independent reviewer was consulted when, if any, uncertainties were identified during the coding and theming processes.

A number of techniques were employed to enhance the rigour of the qualitative data collection, analysis and interpretation processes. A range of strategies were used to promote credibility, transferability, dependability and confirmability of data analysis and interpretation. These included adherence to the semi-structured interview guide, audiotaping interviews, transcribing verbatim by an independent and external typist, use of more than one researcher for the purpose of coding and cross checking between the research team [20, 21]. These processes were familiar to the research team as they had been used previously [16]. All data were de-identified to promote trustworthiness of the analysis process. The research team also regularly consulted with external stakeholders as means of independent verification of data analysis and interpretation. Prior to research commencement, an independent review of the research processes was undertaken by the Human Research Ethics Committee.

### Ethics

Ethical approval was obtained from the Human Research Ethics Committee from the University of South Australia [Protocol number – 0000036445]. As part of recruitment process, participants were provided with Participant Information Sheet which outlined the nature and purpose of the research, the background of the research team and the proposed aims/outcomes of the research. The participants were informed that their participation in this research was entirely voluntary, they could withdraw from this research at any time without any consequences and no incentives were provided for their participation.

## Results

### Overview of participants

In total 22 participants, 16 AHPs and six employers/managers, shared their perspectives on enablers of and barriers to transition to rural practice by AHPs across South Australia. Of the six employers/managers who participated in the semi-structured interviews, three participants were from private sector, two were from public sector, and one participant worked in a non-government organisation (NGO). The health care services were delivered in various regions within South Australia, including Barossa Valley, Eyre Peninsula, Far North, Limestone Coast, Murray Mallee, Yorke and Mid North. One employer reported that their services were provided across different regions. The demographic details are presented in Table 1.

**Table 1:** Overview of Employers

	Number of Employers
Organisations	
Public sector	2
Private sector	3
NGO^a^	1
Service Delivery Regions	
Barossa Valley	1
Eyre Peninsula	2
Far North	1
Limestone Coast	1
Murray Mallee	1
Yorke and Mid North	2

^a^NGO – non-government organisation;

There were 16 AHPs who participated in the semi-structured interviews (Table 2*)*, including three physiotherapists, speech pathologists, podiatrists, and social workers along with one dietitian, occupational therapist, pharmacist and provisional psychologist. At the time of interview, some AHPs (*n* = 3) started working in metropolitan areas, however all demographic data presented in Table 2 was in relation to AHPs’ rural-based positions.

**Table 2:** Overview of AHPs

	Number of AHPs
Allied Health Disciplines	
Dietetics	1
Occupational Therapy	1
Pharmacy	1
Physiotherapy	3
Podiatry	3
Psychology	1
Social work	3
Speech Pathology	3
Organisations	
Public sector *only*	12
NGO^a^	2
Mixed (public and private sectors)	2
Locations (by regions)	
Eyre Peninsula	7
Far North	2
Limestone Coast	3
Murray Mallee	1
Yorke and Mid North	3
Exposure to Transition Programs	
RHPP^a^ *only*	3
TPPP^a^ *only*	8
RHPP and TPPP	5
Length of time in Rural Practice	
One year or less	4
More than 1 year	8
More than 2 years	2
Three years and more	2
Age Groups	
20–30 years	11
30+ years	1
Previous Rural Placements	
Yes	8
No	8
Rural Background/Living Experience	
Yes	10
No	5
Yes, but outside Australia	1

^a^NGO – non-government organisation; RHPP – Rural Health Professionals Program; TPPP – Transition to Rural Practice Program;

Most of these AHPs (*n* = 12) had current or previous employment with the public sector. Additionally, two participants reported past or current work experience across both public and private sectors while another two AHPs were from NGOs. The AHPs worked across five different regions within the State, including Eyre Peninsula, Far North, Limestone Coast, Murray Mallee, Yorke Peninsula and Mid North. Three AHPs had experience working across different regions, however only the region where they worked longest was included in Table 2. All of the AHPs reported participation in at least one transition program. Three AHPs reported exposure to RHPP, eight AHPs were part of the TPPP and five AHPs were involved in both programs.

The length of practice in rural areas was diverse ranging from four months to more than four years. Twelve out of 16 AHPs reported their age with the majority of practitioners aged between 20 and 30. AHPs’ rural experiences, such as their rural placement experience and rural background/living experience were also explored. Half of the AHPs reported previous rural placement experience and many also had rural background or rural living experience.

Thematic analysis of the interview data resulted in a number of key issues impacting transition to rural-based practice. As means of presentation, these findings were categorised into three stages during the transition: ‘***before’, ‘during’*** and ***‘after’.*** The ***‘before’*** stage comprised of issues participants reported prior to the transition to rural-based practice (‘going rural’). The ***‘during”*** stage comprised of issues participants reported during transition including living and working (including placements) in rural areas. The ***‘after’*** stage comprised of issues participants reported once transition had well and truly been completed and related to issues they had confronted while ‘staying rural’. Within each stage, five key themes emerged namely *social/lifestyle; nature of rural practice; mentor and support; exposure to rural ‘taster’* and *job availability/characteristics*. While the findings have been categorised into these stages for presentation and clarity, it is important to recognise that transition to rural-based practice may be more accurately described as a continuum rather than distinct stages. Additionally, it was identified that many barriers and enablers are essentially ‘two sides of the same coin’. Therefore to avoid repetition, results were not categorised into barriers and enablers.

### ‘Before’ stage

#### ‘Before’ stage – nature of rural practice

*Nature of rural practice* was key theme common to both AHPs and employers in the ‘*before’* stage. *Nature of rural practice* related to factors associated specifically to the rural work environment and duties. Under this theme, *variety of caseloads* was an important factor. AHPs discussed that the varied nature of caseloads in rural areas was appealing and a factor to ‘go rural’ (*n* = 7).

*“… I think number one was the variety working in a rural area, so that’s being a generalist clinician, having both adult and paediatric, and a variety of client presentations was a really big drawcard for me…”* **AHP16** (> *1 year rural practice experience).*

Employers also held similar views, highlighting positives of the *variety of caseloads* in rural areas as a ‘selling point’ in attracting AHPs to the country.

“*In actual fact, working rurally is the opposite, I think you get exposed to a wider range of things and it can be challenging but very exciting at the same time. I think we need to sell that to people to make them want to come to country*.” **E5.**

Interestingly, an employer from private practice also mentioned their ability to provide AHPs with *variety* as well as *specialty areas.* This appeared to be in response to AHPs desire to also have the opportunity to specialise in areas which had previously not been a trait of rural practice.

*“…Because some people do want to specialise and I think even though we work generally, I think we need to give people the opportunity to specialise……..So there’s certain things we can do now from a specialty side that we didn’t do in the past, and that’s a massive sell point for us.”* **E5.**

#### ‘Before’ stage – exposure to rural ‘taster’

*Exposure to rural ‘taster’* was a unique theme to the ‘*before*’ stage. The theme was common to both AHPs and employers. It encompassed factors relating to rural experience including rural background/living, rural student placements and rural experience of other people (recommendations to ‘go rural’). The participants exchanged their perspectives on the impact of rural experience to transition to rural-based practice.

In particular, *rural background* was discussed by AHPs in terms of the impact it had on their decision to ‘go rural’. Some AHPs reported that they were from rural areas and always had the intention to return.

*“I’m a country girl originally so born and based in a small rural town, so I sort of always had the intention to go to [metropolitan city] for training and then end up in a country town somewhere along the line…“***AHP7** (*≥3 years rural practice experience).*

Similarly to this, one employer from private practice highlighted offering placements to ‘local’ students as they are more likely to return to rural areas.

*“So it’s huge, and I think that’s really important. We have a bit of a philosophy of prioritising students who are from the local area because we feel that they’re the ones who are most likely to return to the general area, if not to the local area itself.”* **E3.**

Some participants reported mixed feelings of the impact that rural background had on their decision to ‘go rural’. They reported feeling unsure if rural background had an impact or not.

*“Yes and no. I don’t think my time in rural areas before [rural town] was that significant to make a big difference for me wanting to come here or not wanting to come here.”* **AHP11** (*≥3 years rural practice experience).*

Additionally, *rural placements* had a positive impact on AHPs’ decision to work rurally as well as actual practice. They highlighted that rural placement gave them the opportunity to experience what rural practice was like.

*“Having experience in a rural placement was definitely helpful. I think it helped to expand that scope of practice and knowing what’s out there other than just metro services.”***AHP8** (*> 2 years rural practice experience).*

Similarly, from employers’ perspective, some employers highlighted the importance of undertaking rural placements for students to experience rural practice and ‘see for themselves’ what the benefits are.

“*So what we do is we work very hard to enable, if anyone comes out with a student here, we try to offer the best possible student placement we possibly can, so they get exposed to lots of things, they see the holistic view point of working rurally, they can see the opportunity. That’s really important. If they can’t see what the benefit is to them, then they’re never going to compete*.” **E5.**

Additionally, AHPs described seeking opinions and recommendations from friends and colleagues who had previously worked at the rural location or had completed placements. The *experience of other people/recommendations* were enablers for AHPs to ‘go rural’ and all AHPs described positive recommendations surrounding the working environment, health care team and rural work load.

*“Yeah, so my colleague mentioned that there was a good, young team out there that the [discipline] team was quite supportive, and he had been enjoying his time out there.”* **AHP5** (*> 1 year rural practice experience).*

Interestingly, employers also discussed the concept of *experience of other people/recommendations* (*n* = 3)*.* From an employer’s perspective these ‘recommendations’ were used as a recruitment strategy to attract new employees. In particular, the employers described maintaining a ‘positive reputation’ to continuously encourage feedback amongst AHP networks to facilitate recruitment to their rural area.

*“… We got another [AHP] from feedback from someone that did a placement with us and he came for an interview based on that feedback…”* **E6.**

Others, especially from private practices, discussed how their previous negative experience with student placements had discouraged them from taking students in the future. The student having no intention to work rurally was a significant negative for the practice in terms of investing in a student’s development.

*“We, however, we offered a rural placement [year] to a student in our own practice and it didn’t go well.”* **E1.**

#### ‘Before’ stage – job availability/characteristics

*Job availability/characteristics* included factors relating to rural positions. These included the availability of rural roles (‘getting experience’), contracts (length and extensions) and organisational recruiting processes (time consuming to recruit to a position). The *job availability* was an important aspect for AHPs in the ‘before’ stage (*n* = 8).

While one AHP mentioned general availability of a position, two participants highlighted difficulties with employment as a new graduate.

*“… So I guess part of my decision to come out to rural location was that no matter where the job was, I was willing to go there because they are very hard to come by.”* **AHP2** (*< 1 year rural practice experience).*

Some AHPs highlighted the perception of rural positions being *less competitive* (*n* = 3) given that there appeared to be *limited position in metropolitan areas* (*n* = 2).

*“So it was very hard to get a job in the city. It took me a really long time to get any job, which sort of took – I wound up applying for a rural position…“***AHP9** (*> 1 year rural practice experience).*

One employer agreed with this highlighting the need to go rural to get experience.

*“It was quite a big step for them to make the decision to go to the country but of course jobs dictated that as well or not having enough jobs in the city meant that they really needed to get out to rural to get some runs on the board, ours anyway, the ones that we’ve actually employed.”* **E4.**

Interestingly, the employer highlighted *organisational* factors effecting recruiting and AHPs desire to working rurally in the ‘before’ stage. *Recruiting processes* were described as *time consuming* and often hindered the employer’s ability to recruit to a position as well as there being a lack of *funding* (*n* = 1)*.* It was identified that these barriers were ‘out of the employer’s hands’ and related to higher organisational departments.

*“The fact our recruitment processes are long and - although that has improved just recently because we kept complaining about it. It takes us often three months to recruit to a position…”* **E6.**

#### ‘Before’ stage – social/lifestyle

*Social/lifestyle* encompassed factors relating to rural lifestyle and AHPs’ social life such as distance from friends and family and social connection with co-workers. *Social/lifestyle* were factors with the ‘before’ stage only listed by employers. This was discussed as recruiting strategies.

In particular, one employer discussed the importance of *social inclusion: embedded into the community.* This was discussed as a strategy put in place to ensure students felt socially included in the hopes that they will return*.* They discussed this in terms of wanting people to come work for them.

*“…So we do try to support the develop and we try and support it to get them embedded into a community if they’re here for a length of time, so they feel part of where we are, so we can support things outside of work*.” **E5.**

Additionally, having a personality that is likely to ‘fit in’ was also a factor in the ‘before’ stage.

*“…and there’s also, I guess, you need to have somewhat of an adventurous spirit to be prepared to move away from home and an independent sort of spirit to be able to do that...”* **E1.**

#### ‘Before’ stage – mentor and support

*Mentor and support* included factors relating to access to mentorship and support available for rural-based work. In particular, one employer discussed the importance of being able to offer AHPs suitable accommodation. They highlighted previous issues with limited available accommodation.

*“…With this as well, we’ve purchased - the business has a house in [rural town] because we often were coming up against… there’s no houses available…. So we have a really nice house available now that’s partly furnished as well so that it makes it a bit easier, and I think that’s really helpful in terms of being able to attract people to the position.*” **E3.**

Furthermore, employers highlighted *financial incentives through RDWA* as a positive enabler in attracting AHPs to rural areas.

*“At times, money becomes tight, so what has helped a few of the ones that get the 12 month plus contracts is they are entitled to apply for a rural health grant of $10,000 that they can spend on professional development. That’s been very successful in getting people to come to the country.*” **E6.**

### ‘During’ stage

#### ‘During’ stage – nature of rural practice

*Nature of rural practice* was one of the common themes to both AHPs and employers in the ‘*during*’ stage. The two stakeholder groups discussed barriers and enablers in relation to practice context. In particular, resource shortage was highlighted by participants.

Team members/leader on leave, the nature of small teams in rural-based settings, and lack of experienced staff were mentioned by AHPs. Not surprisingly, almost all AHPs felt lack of support as a result.

*“… when I got here my team leader at the time went on extended leave so there was a period about seven weeks I didn’t have a team leader. I found that was a difficult period because being a new graduate I felt like I didn’t have that support that I needed from a team leader because there was no team leader…“***AHP1** (*< 1 year rural practice experience).*

This finding was supported by an employer from public sector. The employer further explained how staff shortage could impact on AHPs, especially new graduates.

*“The other barriers would be if I’m short of staff to start off with - so if I’ve not been able to recruit to positions and we get a newbie in, then they don’t always feel supported. We had one situation…we had a [AHP] that quit [the profession] because of her experience working with us…because she didn’t feel supported…because of lack of senior staff around, they can come into a discipline where, if they don’t get the guidance, then they struggle. Whereas in a larger situation or a larger hospital, there’s always a senior around.”* **E6.**

In addition to staff shortage, another employer discussed other resource barriers, which also linked to limited provision of mentoring/support to AHPs. Lack of dedicated roles to provide mentoring/support was one barrier highlighted by the employer.

*“… We struggle continually resource wise….we’re painfully aware that because the people that are actually providing the mentoring and supervision are also service delivery people. We don’t have dedicated roles to provide this, they also carry a case load as well so it’s quite challenging as a lean organisation to provide adequate levels of mentoring and supervision. It’s definitely one of our challenges.”* **E4.**

Employers highlighted other issues in relation to practice context. Three employers from private sector mentioned that geographical location was a barrier to physically accessing professional development (PD).

*“…And even in terms of us being able to access training and development in our own state there’s almost none for that cohort…we are working with. The training we can access in South Australia doesn’t match the severity of the [patient cohort] that we’re seeing, it does occasionally but most of the time not, so often having to go interstate for training as well.”* **E1.**

While recognising this issue, the same employer discussed strategies within their organisation to overcome the geographical barrier and maximise support for AHPs.

*“…so I guess that’s why [person’s name] puts in a big effort to ensure that they have regular PD sessions about every six weeks, and encouraging people to go and attend different seminars that go on at different times, so they can share that knowledge. So one person goes and everyone benefits from it. And that’s how it goes*.” **E2.**

Another employer discussed the development of networks in rural areas as a strategy to avoid professional isolation for AHPs.

*“..We also have discipline networks, so the whole of country at least come together twice a year, so you can cross pollinate and network with people from all over the [organisation].”* **E5.**

*Model of care* was another area within practice context discussed by some employers. They specifically highlighted that AHPs are usually the *primary contact practitioner* in rural areas, thus they are expected to make important and critical decisions immediately. These were perceived as a barrier by the employers.

*“I think professionally working in a rural area, although very challenging…there is huge expectations on a diagnosis in the country and certainly you’re often seeing people as a primary contact practitioner and often patients come to see us without a referral and they’ve never been to a doctor in their life...”* **E3.**

#### ‘During’ stage – social/lifestyle

*Social/lifestyle* was another common theme discussed by both AHPs and employers in the ‘*during*’ stage. The participants exchanged their views on both barriers and enablers that were related to AHPs’ social life. Distance was mentioned by AHPs as being away from family and friends was regarded as a factor that hindered their transition.

*“I think on a personal level being away from family and where I grew up – it was the first time I had moved out of home and moved out from mum and dad, so that transition didn’t really hit me until the end of my first year like missing being close to family and the convenience of having family and friends and a familiar environment.”***AHP6** (*> 1 year rural practice experience).*

Many AHPs discussed social inclusion as an enabler in the ‘*during*’ stage, they particularly highlighted that establishment of social networks at the workplace facilitated their transition.

*“…I think things that facilitate the transition are good access to a social network in the work environment so co-workers and colleagues that are in a similar position to you to help you feel a part of the team and a part of the community...”* **AHP5** (*> 1 year rural practice experience).*

Being able to socially connect with co-workers was also identified as an important enabler by two employers. The employers specifically discussed how their team built social relationship outside working environment.

*“… Most of my team are young…So I think actually that helps as well because people then don’t feel socially isolated…..But my team are so supportive, when they get a new one, it wouldn’t matter what discipline it was in; we always have a welcome lunch. They always feel welcome. So that kind of supportive environment helps graduates.”* **E6.**

Embedding AHPs into local community was recognised as a facilitator in both ‘*before*’ and ‘*during*’ stages. The employers previously discussed it as a recruitment strategy to attract AHP’s to ‘go rural’.

Employers also highlighted reasons which negatively impact on AHPs’ ability to socially embed into the community. One employer perceived that frequent travelling back home was one barrier.

*“What we haven’t really had is …the staff will go home on the weekends and none of them have gotten involved in the community which has been disappointing because I think that would be a really great thing…”* **E1.**

#### ‘During’ stage – job availability/characteristics

The theme of *job availability/characteristics* was specifically discussed by employers from private sector. One employer indicated the importance of demonstrating the change of career progression within their practice, which might potentially facilitate AHPs transition.

*“And then I think seeing some sort of career pathway as well…If we have a new graduate they can see in 10 years’ time that they might be able to be in that senior [the profession] position.****”*** **E3.**

Another private practice employer discussed generous remuneration and earning a guaranteed wage plus commission. They explained this as having the ability to earn more while also having the ‘security’ of a minimum wage.

*“…It’s about guaranteeing minimum earnings as well as paying percentages on the consultation…It actually just gives them that confidence and a little bit of security to know that they’re going to earn that as a minimum.”* **E2.**

#### ‘During’ stage – mentor and support

*Mentor and support* was an important theme discussed by both AHPs and employers within the ‘*during*’ stage. In particular, employers discussed parameters, processes and structures (e.g. financial and accommodation) required for support and mentoring. On the other hand, barriers and enablers to transition were highlighted by the AHPs.

Within this theme, the mentorship and support was discussed from the employer’s perspective. The employers mainly discussed *what* they were providing and *how* they were providing it. Therefore this section was catagorised into *structures (*e.g. *accommodation and financial supports), parameters (*e.g. *duration and type of support)* and *process (how the support is provided* e.g. *frameworks).*

##### Structures of support

Many of the employers discussed various structures utilised to provide support for AHPs. Various financial supports were utilised in a number of areas. In particular, financial supports for PD were an important factor discussed by employers. Different sources of funding/finance were drawn on to provide this support.

Three employers reported providing access to company grants for AHPs to use towards PD and associated costs such as travel and accommodation. These employers were from the private sector or NGOs.

*“So all of our staff we give access to [dollar amount] and two day’s paid leave per year to attend continuing professional development.”***E1.**

Additionally, paid external supervision was another financial support provided by one employer.

“*We pay for external supervision. There are a number of our staff who undertake external supervision. That’s something that we also pay for…”* **E4.**

Although not specifically financial support, one employer from the public sector discussed being supportive of all PD requests placed by AHPs. They discussed this in terms of encouraging attending PD as well as approving nearly all requests.

“*… The other things we do here is we do strongly promote professional development….we don’t quibble about that because we think that supports graduates. It’s great to do a university course, but sometimes, once you’ve started, you identify an area that you need to go into. So we’re very supportive of that.”* **E6.**

##### Parameters of support

The employers discussed various *parameters of support* provided. In particular, they mentioned the support person(s) involved in providing mentoring and supports to the AHPs. From an employer’s perspective many people were involved. For example, senior practitioners, discipline specific teams, multidisciplinary teams and multidisciplinary team leaders were all identified as support person(s).

Only the employers within the public sector discussed having a specific ‘clinical supervisor’. This was discussed as part of clinical supervisor framework. It was also mentioned that this supervisor may be ‘offsite’, however this was not ideal and should be avoided if possible.

“*…We’ve also got a clinical governance framework in place that - they’ve all got a clinical supervisor. That might be off site. My staff are very good at knowing that they can go to that clinical supervisor if it’s a clinical issue they think they can’t resolve themselves. So that does support them as well.*” **E6.**

The frequency and duration of supervision varied between settings. Most discussed meetings varying from weekly to monthly, lasting for approximately one to two hours. Nearly all employers (*n* = 3) highlighted that the AHPs were ‘never working alone’ and that senior staff were available to answer questions.

##### *Proces*ses *of support*

Various processes of support were discussed by employers, in terms of how the support was provided. Employers within the public sector highlighted specific frameworks in place to assist with the provision of appropriate supervision. One employer in the private sector highlighted structured PD sessions. Another employer in the NGO sector discussed the process of developing an organisational supervision framework. One employer in the private sector offered a specific new graduate training program to AHPs during their first year. Additional processes such as network connections and linking with other organisations were mentioned by one employer. This enabled the employer to link with other organisations to provide PD in specific areas.

##### AHPs perspectives

Given the various mentorship and support offered by employers, it was not surprising that many AHPs valued the different types of support they received from several sources. There were critical factors which positively or negatively influenced their transition to rural-based practice.

Supportive team and co-workers was a major enabler discussed by many AHPs. This was different to previously discussed social connections with co-workers under *social/lifestyle*, as AHPs described supports received from their teams/co-workers which facilitated their transition into the workplace. Some AHPs also mentioned supports provided by their seniors or supervisors, which facilitated their transition.

*“And I’ve been really fortunate that my senior in my department here in [rural town] being really lovely and welcoming and basically [the team] in general have been really welcoming and supportive and made the transition really good.”* **AHP6** (*> 1 year rural practice experience*).

Financial support from external agencies was another enabler. Of the people who received specific transition packages (such as RDWA packages), funding was perceived as extremely helpful by participants. Moving expenses were deemed very expensive by AHPs and scholarships relieved some of the financial pressures or prevented ‘putting them behind financially’.

*“Knowing that I had some finance around setting me up certainly helped…. And I may have even said it’s too hard. And you know, even though this is my dream job and everything else, I may have got a bit of cold feet.”* **AHP4** (*< 1 year rural practice experience).*

Staff shortage leading to lack of support for new AHP’s was a barrier to transition to rural practice in the ‘*during*’ stage. As this issue linked with the *nature of rural practice*, it has been discussed previously.

### ‘After’ stage

#### ‘After’ stage – nature of rural practice

Both AHPs and employers shared their perspectives on barriers and enablers once the transition to rural-based practice had occurred. Within this, *variety of caseloads* was a factor which was commonly discussed by both groups. AHPs highly valued the range of conditions and patients they had seen in rural areas and many agreed that such diversity was a main reason for them to ‘stay in rural’.

*“The caseload here has been fantastic and I have varying days and I haven’t really lost any of the skills I’ve learnt at university. I really also like the idea of becoming quite experienced quite quickly and that ability to be reclassified much sooner than say in a metro setting…”* **AHP6** (*> 1 year rural practice experience*).

Employers also highlighted that the positive impact of rural practice on local community was another factor which attracted AHPs staying and continuously working in rural areas.

*“But the people they’re working with are so rewarding to treat, and it’s why I continue to live and work down here”* **E3.**

However, there were negative features to rural practice, mainly in the form of staff shortages. Not only newly employed AHPs felt unsupported and eventually left, lack of staff also placed additional burden on current staff members. Thus they moved to other areas as they felt overworked, stressed and not supported.

*“If you’re short of staff to start off with and people are feeling stressed because they feel like they have to work harder…So people move onto areas that have more staff, so they feel more supported. That’s a significant barrier.*” **E6.**

#### ‘After’ stage – social/lifestyle

The issue of *social/lifestyle* was commonly reported by both AHPs and employers. Geographic location, country community and lifestyle were the major factors reported by AHPs to ‘stay in rural’.

*“Being able to live in a town which is near the [attraction] and it’s a bit of a gateway to a beautiful part of the state for weekend adventures and trips out into the [attraction] and camping trips…”* **AHP5** (*> 1 year rural practice experience*).

Socially embedded into the local community was another reason for many AHPs to retain in rural areas. In particular, they highlighted the formation of social networks and friendships with local people and groups.

*“… and of course the friendships I’ve made…it’s that nice sense of belonging and having really good friends; That’s also another reason to stay…”* **AHP16** (*> 1 year rural practice experience*).

While many AHPs successfully built up local connections and remained in rural areas, there were some AHPs who had left due to distance away from family, friends and relationships, which appeared as a barrier.

*“It’s a bit isolating up there. All my friends were also back here and while the people I worked with were good, it wasn’t the same. You didn’t have that support network that you do have when you’re back home.”* **AHP9** (*> 1 year rural practice experience*).

This was also identified by employers. They further commented that having personal relationships developed in rural areas would probably facilitate AHPs to stay.

*“…– the social stuff is the stuff that’s - so our [AHP] moved backed to [Metropolitan city] because she has a relationship with someone back there and he wanted her closer to home”* **E1.**

#### ‘After’ stage – job availability/characteristics

*Job availability/characteristics* was another commonly discussed issue by both AHPs and employers. They specifically commented on barriers relating to the nature of rural employment. One AHP perceived the length and availability of contract contributed significantly to staying within rural-base settings.

*“… I think it comes down to employment opportunities really, because my current role is a contract role and I know that it’s only funded for a certain amount of time but there’s not really any availability in other areas…”* **AHP12** (*> 1 year rural practice experience*).

Another AHP who left rural workplace agreed and reported that the length of contract played a role in leaving the previous rural-based position.

*“Half way through my work in [rural town], I was offered a job interview for [organisation], and for various reasons - it was pay, length of contract and being able to be close to family back in [city]…”* **AHP10** (*> 1 year rural practice experience).*

This was also echoed by employers from different sectors. One employer from public sector mentioned that short-term contracts were an issue in keeping AHPs in rural areas. The employer highlighted that their ability to extend contracts was very limited.

*“What hinders it is the short term nature of contracts, which we’ve got no control over because it’s governed by the funding model. Not the ability to extend those contracts...”* **E6.**

In contrast, as employers in private sector had the ability to extend some contracts, they perceived contract as “*an incentive”* to retain AHPs in rural workforce.

*“… But it’s a minimum of a 12 month commitment that we expect from them, and we intend to offer them a second year contract as a minimum if they’re interested in staying on, I guess as a bit of an incentive, you know, that it’s a longer term position. It’s not just a 12 month grad placement.”* **E3.**

#### ‘After’ stage – mentor and support

The issue of *mentor and support* was also discussed by AHPs and employers in the ‘*after*’ stage. Similar to ‘*during*’ stage, many AHPs highlighted supportive team and co-worker as an important enabler for them to ‘stay in rural’.

*“I think a lot of it has been the workplace culture…. actually really enjoying going to work and getting to work with a really great supportive team which have open communication, open to new ideas, that has been something that obviously you want to keep because finding that is quite rare…”* **AHP16** (*> 1 year rural practice experience*).

Employers reflected on their strategies which they believed help to retain AHPs. Having a supportive environment was discussed as a facilitator to keeping staff by one employer.

*“…I think what facilitates us keeping staff is the supportive environment. We’ve had so many short term contracts that would love to have stayed with us but we couldn’t offer them anymore. That tells me we’ve got a reasonable environment for people to work in.”* **E6.**

Other employers went on to discuss specific support strategies they had put in place. These included learning plans (“*we try and formulate a plan around that each year so they hopefully develop into competent and excellent senior clinicians”* **E5**)**,** structured support (“*we’ve got quite a structured professional development programme for everybody, not just for the new grads”* **E3***)* and multifactorial incentives (“*we can offer her more autonomy, good supervision, good access to CPD. We treat her really as a member of the family”* **E1**)*.*

## Discussion

The growing chasm between metropolitan and rural health care requires immediate attention and prompt action. While much of the attention to date has been on increasing workforce supply, through strategies such as improved remuneration, this has not resulted in sustainable positive impact. In order to tackle this challenge, it is imperative to view the transition from metropolitan to rural practice as a continuum and understand what facilitates and hinders this transition. This research explored the enablers of and barriers to transition to rural practice by AHPs in South Australia from different stakeholders’ perspectives.

A range of barriers and enablers were identified and were grouped across five overarching themes – *nature of rural practice, exposure to rural ‘taster’, social/lifestyle, job availability/characteristics,* and *mentor and support.* In particular, *exposure to rural ‘taster’, social/lifestyle,* and *mentor and support* were the key themes reported by the stakeholders. The multifactorial nature of the barriers and enablers highlight the complexity underpinning how AHPs transition to rural-based practice. These barriers/ enablers are often inter-linked and continually evolving which pose significant challenges for health care stakeholders to successfully address these.

Findings pertaining to the critical role *social/lifestyle* factors played during transition are supported by previous research findings by Campbell, McAllister & Eley [14]. The research by Campbell and colleagues identified that rural lifestyle, family, friendships and community connectedness were critical motivators for AHPs to work in rural settings. Similarly, this research identified the importance of social inclusion of AHPs within rural communities, which was addressed by the employers through targeted strategies. Employers who participated in this research were practitioners and/or managers with many years’ experience. It is likely that over the years they have identified social/lifestyle aspect as a critical factor to assist the transition and to retain employees. The importance of embedding AHPs within the rural community has also been previously recognised as a critical factor [22]. This is an important finding as it highlights that for successful transition to rural practice *social/lifestyle* factors need to be carefully addressed. This research identified that being away from family and friends was a significant barrier, especially for those in a personal relationship, and evidence from other research supports this finding [23].

*Exposure to rural ‘taster’* was a standout finding for both AHPs and employers. Rural experience through placements and background/living had positive impacts on AHPs’ decision to ‘go rural’ and transition to rural practice. This finding is consistent with other literature which highlighted that AHPs with rural backgrounds were often attracted to work rurally [24]. Other literature also supports that rural placements are also strong predictors of ‘going rural’ [25]. Given this consistent finding, it is critical that rural placements may act as fertile recruiting grounds, provided they are positive, as students return or give positive feedback about the rural areas to their peers [23]. While it is important to recognise the positive impacts of rural placements, this research also identified some hesitation from employer’s perspective, especially in the private sector. These findings are supported by Shannon et al. [26] who identified financial burden and time constraints as barriers to having students in rural areas.

Another finding was the critical role of, and impact from, *mentor and support* in rural practice. Limited availability of mentorship and support, such as through clinical supervision, in rural and remote areas for AHPs has been well documented in the literature [27–29]. Lack of mentorship and support was specifically linked to staff shortage in this research, which has also been documented previously in the literature [30]. Health professionals including AHPs practising in non-metropolitan areas rely upon supervision and mentorship as part of professional support [16]. This type of support involves provision of professional education and training by approved supervisors such as seniors or experienced staff [16, 31]. Clinical supervision is critical in improving health care quality as it provides benefits to health professionals [32, 33], patients [34, 35] and organisations [36]. Given this to be the case, the limited availability of mentorship and support for AHPs in rural practice, over time, resulted in some of AHPs leaving rural-based practice. In contrast, AHPs in this research highlighted that supportive team and co-workers were a key factor which facilitated their transition to rural practice. Many of them further emphasised that working within a supportive team was also a reason to ‘stay in rural’. This finding is consistent with previous research as teamwork is recognised as one of the key valuable aspects working and remaining in rural and remote areas [28, 37, 38].

The findings from this research indicate that some incentives such as scholarship and availability of accommodation were factors they perceived as attracting AHPs to ‘go rural’. These findings are supported by previous research by Gillham and Ristevski [23] but also caution that financial incentives alone are not an important retention factor. Keane and colleagues [24] also acknowledged that financial incentives were regarded as lower priority compared to other factors such as access to continuing professional development (CPD) in rural practice.

### Limitations

As with any research, this research too has some limitations. Firstly, despite several attempts, in terms of recruitment of participants, there was imbalance amongst the AHPs (three physiotherapists versus one dietitian). Second, this research was conducted within one geographical location in Australia (South Australia). While this may limit the transferability of these findings to a range of other contexts, they nevertheless provide some useful insight into transition issues that require ongoing exploration and research. Finally, while this research provides rich information about transition to rural practice from AHPs and employers perspective, it does not provide what occurs from an allied health student perspective. Further research with this stakeholder group is required.

## Conclusions

The findings from this research contribute to the growing evidence base for best practice transition in allied health. The findings from this study indicate that a number of factors play a critical enabling or hindering role to transition to rural practice by AHPs. These factors are complex, do not operate in isolation and are often interlinked. Factors such as exposure to rural ‘taster’, social/lifestyle, and mentorship and support can considerably impact transition to rural practice by AHPs. While incentives such as financial and accommodation supports were welcomed, they do not appear to play a casual role. With increasing focus on closing the chasm between metropolitan and rural health care, it is imperative that strategies which promote transition to rural practice are underpinned by current best evidence regarding “what works” at the coal-face. The findings from this research provide important lessons for successful and sustainable transition to rural practice by AHPs.

## Data Availability

To maintain the privacy of study participants who work or previously worked in small rural towns across South Australia, the qualitative data generated and analysed during the current study are not publicly available. However, the datasets are available from the corresponding author on reasonable request.

## Abbreviations

AHP(s): Allied health professional(s)
CPD: Continuing professional development
NGO: Non-government organization
PD: Professional development
QD: Qualitative descriptive
RDWA: Rural Doctors Workforce Agency
RHPP: Rural Health Professionals Program
TPPP: Transition to Rural Practice Program
